# Primary Sclerosing Cholangitis: Diagnosis, Management, and Clinical Challenges

**DOI:** 10.3390/jcm15031149

**Published:** 2026-02-02

**Authors:** Sofia Svensson Di Giorgio, Chiara Maria Scandavini, Antonio Molinaro, Urban Arnelo, Roberto Valente

**Affiliations:** 1 Department of Diagnostics and Intervention, Surgery, Umeå University, 901 87 Umeå, Sweden; sofiasvenssondigiorgio@gmail.com (S.S.D.G.); chiara.scandavini@regionvasterbotten.se (C.M.S.); urban.arnelo@umu.se (U.A.); 2 Department of Molecular and Clinical Medicine, Wallenberg Laboratory, University of Gothenburg, 405 30 Gothenburg, Sweden; antonio.molinaro@wlab.gu.se; 3 Department of Surgery, Division of Surgical Oncology, University of Colorado School of Medicine, Aurora, CO 80045, USA

**Keywords:** sclerosing cholangitis, PSC, cholangiocarcinoma, diagnosis, clinical challenges

## Abstract

Primary sclerosing cholangitis is a rare, chronic, inflammatory disease of the biliary tree that leads to progressive ductal obliteration, hepatic dysfunction, and ultimately liver cirrhosis. Most patients eventually require liver transplantation or develop serious complications, the most severe being end-stage liver disease and cholangiocarcinoma. The global prevalence of PSC is rising and has been reported to reach up to 31.7 cases per 100,000 individuals, representing a significant challenge in both diagnosis and management. In this review, we aim to provide a clinically oriented overview of the diagnosis and management of PSC. Furthermore, we seek to highlight key challenges and areas of uncertainty that clinicians encounter in the diagnosis and treatment of patients with this complex disease.

## 1. Introduction

Primary sclerosing cholangitis (PSC) is a rare, chronic, inflammatory disease that affects the biliary tract, leading to progressive destruction of extrahepatic and intrahepatic bile ducts. This translates into liver dysfunction, cholestasis, and eventually cirrhosis [1]. Most patients develop end-stage liver disease and require liver transplantation [2]. The global prevalence of PSC can be as high as 31.7 per 100,000 individuals, although prevalence varies significantly worldwide, with the highest rates reported in northern Europe (particularly in the Scandinavian region) and a lower incidence in southern Europe and the Middle East [3,4,5].

In addition, other autoinflammatory conditions may co-occur in patients with PSC [6,7], including inflammatory bowel disease (IBD), in up to 80% of cases [7]. The coexistence of IBD and PSC is higher in Scandinavian countries compared to southern Europe. Nevertheless, this difference may reflect selection bias, as Scandinavian countries have easier access to colonoscopy and therefore a lower rate of undiagnosed IBD. In general, the prevalence of PSC in patients with IBD is around 2%, and it correlates with the extent of colitis, being higher in cases of pancolitis [8].

Although symptoms may significantly impact patients’ quality of life, the role of PSC in the development of subsequent cancer warrants particular attention. Indeed, PSC is considered to be the major risk factor for hepato-pancreato-biliary cancers (HPB-cancers), and the risk of cholangiocarcinoma is approximately 400-fold higher that observed in the general population [9,10,11]. Up to 50% of patients with cholangiocarcinoma were diagnosed within the first year after the PSC diagnosis, while the lifetime cumulative incidence is as high as 20% [9,12,13,14,15,16]. In addition to cholangiocarcinoma, PSC harbors an increased lifetime risk for the occurrence of hepatocellular carcinoma (HCC), gallbladder carcinoma, pancreatic cancer, and colorectal carcinoma [17,18,19]. Although follow-up cross-sectional abdominal imaging with magnetic resonance imaging (MRI) has been shown to reduce mortality by approximately 71%, this effect appears to be more related to the earlier identification of conditions amenable to endoscopic treatment, rather than a direct impact on mortality after the diagnosis of cholangiocarcinoma [20].

## 2. Materials and Methods

This narrative review was based on a PubMed literature search. The search strategy combined terms related to “primary sclerosing cholangitis” OR “PSC” OR “sclerosing cholangitis” AND terms covering the main clinically oriented domains addressed in this review (such as “diagnosis”, “imaging”, “endoscopy/ERCP”, “MRI”, “cancer surveillance”, “prognostic tools”, “stricture”, “transplantation”, “machine learning”, “artificial intelligence”). Noteworthy, those research terms have been screened with different combinations but not through a systematic search. Moreover, relevant publications have been added my manual search through bibliographies. Only English-language articles with available full text were considered. We also reviewed various sources, including original articles, reviews, and other types of publications that allowed for a more in-depth investigation of the topic. In addition, a bibliographic search of the references was performed to identify any articles that may have been missed by the initial search string.

Artificial-intelligence–assisted language editing (ChatGPT 5.2, OpenAI) was used to refine grammar and style under the direct supervision of the authors, who take full responsibility for the content. ChatGTP was used to modify the image created on Bio Render.

## 3. Results

### 3.1. Signs, Symptoms

**Figure 1:**
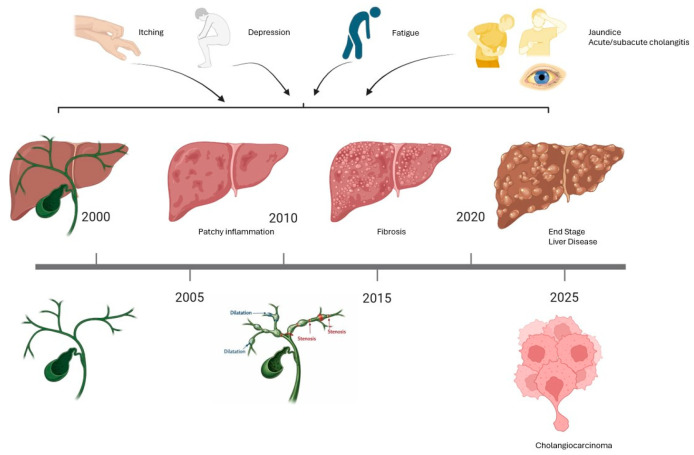
Signs, symptoms, and evolution of PSC.

Clinically, PSC can manifest through a range of signs and symptoms, including abnormal liver tests, weight loss, fatigue, right upper quadrant pain, jaundice, pruritus, cholangitis, portal hypertension, or cholangiocarcinoma [21,22]. Approximately 50% of patients with PSC are asymptomatic at the time of diagnosis. When retrospectively evaluating the possible presence of symptoms prior to diagnosis, about ¾ of cases report abdominal pain, itching, and fatigue, while ¼ of patients report depressive-type symptoms. Abdominal pain and itching are often attributable to bile duct strictures, and when fever is also present, the possible onset of acute bacterial cholangitis should be considered, based on the obstruction or stenosis of the bile ducts (Figure 1) [23].

### 3.2. Diagnosis

#### 3.2.1. Nomenclature and Diagnostic Criteria

Recently, the International PSC Study Group redefined the criteria for identifying possible dominant strictures when the following criteria exist before endoscopic retrograde cholangiopancreatography (ERCP) [3]:

1. Radiological Evidence: A stenosis observed on MRI or ERCP, characterized by narrowing of the major bile ducts, with or without upstream dilation.

AND

2. Symptoms and biochemistry impairment: obstructive jaundice, cholangitis, or pruritus AND an increase in bilirubin and alkaline phosphatase (ALP) levels exceeding 1.2 times the upper limit.

OR

Biochemistry alone: worsening of bilirubin and ALP levels over the preceding six months, reaching at least 1.5 times the baseline value or double the upper limit.

Dominant strictures are defined as definitive dominant strictures when additional criteria are met [3]:

- Radiological, Biochemical, and Symptomatic Indicators: As outlined above.

AND

- ERCP Findings: Difficulty in passing a catheter with a diameter greater than 5 Fr.

OR

- Treatment Response: Symptomatic and biochemical improvement following stent placement or dilation, typically assessed approximately as 20% improvement of ALP/bilirubin two/four weeks after ERCP.

In 2023, the American Association for the Study of Liver Diseases (AASLD) revised its guidelines, significantly modifying the terminology by introducing the concept of a clinically relevant stricture. This is defined as a stenosis of the common hepatic duct or its first-order intrahepatic branches, associated with obstructive symptoms or the occurrence of bacterial cholangitis. It is noteworthy that this definition closely resembles the previously used term possible dominant stricture, and, in any case, such a diagnosis can only be established following the execution of an ERCP [3].

The dominant strictures are regarded as a possible malignancy marker [24]. In fact, cholangiocarcinoma often arises at the site of dominant strictures [15]. According to the 2009 EASL Clinical Practice Guidelines, PSC should be suspected in patients with elevated serum markers of cholestasis, i.e., ALP and gamma-glutamyl transferase (GGT), and bile duct irregularities on MRCP or ERCP, once secondary causes of cholestasis are excluded [25]. Given that cholangiocarcinoma often occurs within the first year after PSC diagnosis, intensive monitoring is crucial. The EASL recommends regular surveillance with yearly “ultrasound and/or MRI surveillance, with or without CA19-9 testing, with modifications according to relevant risk factors” [26].

#### 3.2.2. Laboratory Tests

A total of 25% of PSC cases present with normal laboratory liver function [21]. Typically, PSC presents with a cholestatic pattern, most commonly featuring elevated ALP and GGT. Serum bilirubin may also be elevated in 28–40% of cases, and aspartate aminotransferase (AST) and alanine aminotransferase (ALT) are often mildly raised (with AST predominance sometimes suggesting cirrhosis) [27,28,29]. Bilirubin tends to rise as the disease progresses [21].

Elevated immunoglobulin M (IgM) levels can be observed [30,31].

There are no disease-specific antibodies for PSC [22]. However, many have been studied.

For example, antineutrophil cytoplasmic antibodies (ANCA) have been detected in patients with PSC, regardless of concomitant IBD [32,33,34,35].

In patients with PSC, ANCA detected by indirect immunofluorescence were not clearly associated with particular clinical characteristics, while antibodies to bactericidal/permeability-increasing protein were found in almost half of the patients [36].

Using indirect immunofluorescence on ethanol-fixed neutrophils, ANCA with a perinuclear staining pattern (p-ANCA) can be detected [37,38].

In PSC/IBD, the predominant pattern is often referred to as “atypical p-ANCA” (also termed p-ANNA) and is typically negative on MPO-ANCA/PR3-ANCA antigen-specific assays, targeting antigens at the nuclear periphery/nuclear envelope [39,40,41].

Methodological aspects matter: on formalin-fixed neutrophils, classical MPO-associated p-ANCA tends to shift to a cytoplasmic pattern, whereas atypical p-ANCA may retain a perinuclear/nuclear-rim pattern [38].

Atypical p-ANCA have been found in up to 70% of patients with PSC, but are not disease-specific [39,42,43]. This subset of p-ANCA have also been called peripheral anti-neutrophil nuclear antibodies (p-ANNA).

Although their high prevalence makes them supportive serological markers, they are not useful for disease monitoring/management, as titers do not reliably correlate with disease activity [44].

Anti-Saccharomyces cerevisiae antibodies (ASCA) have also been reported in PSC (e.g., 30% and 53% in two studies), but they lack disease specificity and can be detected in other immune-mediated conditions such as coeliac disease [40,45].

In PSC, neither ASCA nor p-ANCA positivity has been consistently associated with clinical/biochemical features, nor with underlying inflammatory bowel disease [45].

The diagnosis of PSC is based on the absence of any identifiable secondary causes. Secondary causes include, among others, cholangiocarcinoma, IgG4-related PSC (IgG4-SC), and choledocholithiasis [22]. IgG4-related PSC may be difficult to distinguish, but is most commonly found in older men, with a history of chemical exposure, and its diagnosis can be made using the HISORt criteria. The HISORt criteria are based on histological, imaging and serological (IgG4) findings, other organ involvement, such as, but not limited to, autoimmune pancreatitis or sialadenitis, and response to corticosteroid treatment [46,47,48]. Nonetheless, elevated IgG values are also observed in patients with a final diagnosis of PSC [49].

To account for all potential differential diagnoses, clinicians typically assess antinuclear antibodies (ANA), antimitochondrial antibodies (AMA), smooth muscle antibodies (SMA), HIV serology, serum angiotensin-converting enzyme (ACE), total immunoglobulins, and immunoglobulin subsets (including IgG4) [22]. The coexistence of an increase in transaminase levels greater than five times the upper limit and an increase in IgG levels greater than two times the normal limit requires consideration of the possible coexistence of autoimmune hepatitis, which is present in approximately one-third of pediatric cases and about 10% of affected adults [3,50,51,52]. Beyond cases where there may be an overlap with autoimmune hepatitis in PSC, the diagnostic value of antibodies particularly anti-neutrophil cytoplasmic antibody (ANCA) and anti-glycoprotein 2 (GP2) is limited and remains an active area of research [3,26,53].

#### 3.2.3. Histopathology

In terms of liver histopathology, PSC is often marked by “onion skin” periductal fibrosis (though this classic lesion may be absent in small-duct PSC), bile duct proliferation, and chronic periportal inflammatory changes [30]. From a morphological perspective, follow-up imaging in patients with PSC typically reveals multiple strictures throughout the biliary tree. Strictures often cause progressive, segmental loss of liver function, recurrent cholangitis, and can hide dysplastic or neoplastic epithelium [54]. Liver biopsy, however, is not routinely performed unless there is suspicion of an overlap syndrome, small-duct PSC, or an unclear diagnosis [22]. Moreover, because percutaneous liver biopsies usually sample only the small, most peripheral portal tracts, the histologic picture in PSC may be entirely normal or show only indirect features of large-duct obstruction [55].

#### 3.2.4. Pathophysiological and Molecular Mechanisms

The etiology remains incompletely understood, nonetheless many theories have been suggested, none of which are mutually exclusive, including the leaky-gut hypothesis and the toxic bile hypothesis [4,23].

The former states that due to inflammation, bacteria and their components translocate from the gut via the portal system [56]. The latter states that abnormal bile composition and dysregulation of bile acid homeostasis might play a role [23,57]. Cholangiocytes are central because together with immune cells, hepatic stellate cells, and portal myofibroblasts, they lead to portal inflammation and periductal (onion skin) fibrosis [58,59].

Another molecule increased is the soluble ICAM-1, which shows expression on proliferating bile ducts in late stage [60,61]. Additionally the bile acid receptor TGR5 (GPBAR1), found on biliary epithelial cells where it promotes secretion, proliferation, and tight junction integrity, is reduced in patients with PSC [62].

Recent clinical data suggest that bile acid accumulation in advanced PSC is accompanied by feedback suppression of bile acid synthesis. In two independent PSC cohorts, the bile acid synthesis marker C4 was inversely associated with total bile acids, and lower C4 independently predicted reduced liver transplantation-free survival [63,64].

PSC is likely to be caused by the interplay of multiple genetic variants, specifically some HLA variants have been identified in a population group, and environmental factors [65,66,67].

A comprehensive discussion of pathophysiological and molecular mechanisms is beyond the scope of this clinically oriented narrative review.

#### 3.2.5. Non-Invasive Imaging

Magnetic resonance imaging with cholangiopancreatography (MRI-MRCP), including three-dimensional imaging and delayed contrast-enhanced T1-weighted sequences in addition to conventional T2-weighted scans, can further improve diagnostic accuracy and can be used to monitor the clinical course of the disease [68]. Today, magnetic resonance cholangiopancreatography (MRCP) is the British Society of Gastroenterology’s recommended imaging modality for visualizing PSC-related biliary changes, largely due to its non-invasive nature and high sensitivity and specificity, of 0.86 and 0.94, respectively [22,69].

MRI-MRCP is particularly useful to identify strictures that need to be further investigated through ERCP [24]. However, cholangiography alone does not reliably distinguish PSC from IgG4-SC or cholangiocarcinoma. Even though the large duct subtype is most common, other variants such as small-duct PSC remain important considerations [21,70]. Small-duct PSC is a recognized variant of the disease (comprising about 5% of cases), and is characterized by a cholestatic biochemical profile and PSC-like histological changes, but with a normal cholangiography appearance [71].

Recently, the ESGAR consensus statement on MRI on PSC confirmed that MR with MRCP is the recommended imaging modality in patients with suspected or confirmed PSC, with annual follow-up, and should be performed before any interventions and biliary stent placement. It provides recommendations on the management of patients, including how to report findings in the biliary tree, spleen, pancreas, and liver [72].

Based on MRI, several scores can be applied. The ANALI score, which can be applied with or without gadolinium, incorporates features such as biliary dilatation, irregularity, and signs of chronic liver disease, and has been shown to correlate with clinical outcomes and transplant-free survival [73]. The DiStrict score instead, derived from MRCP, quantifies PSC severity based on the extent and degree of intrahepatic and extrahepatic ductal changes [74]. Finally, GLRLM-Run Entropy in FS-T2W was developed for risk stratification [75].

Recommendations include regular liver stiffness measurement (LSM) with Vibration-controlled transient elastography (VCTE) (e.g., FibroScan^®^, an ultrasound-based elastography method) to study the degree of liver fibrosis [76].

VCTE is able to differentiate severe from non-severe liver fibrosis with high levels of confidence in patients with PSC, in fact LSM was independently linked to the stage of fibrosis: “cutoff values for fibrosis stages ≥F1, ≥F2, ≥F3, and F4 were 7.4 kPa, 8.6 kPa, 9.6 kPa, and 14.4 kPa, respectively”. Baseline measurements and rate of LSM progression were strongly and independently linked with patients’ outcomes [77].

#### 3.2.6. The Role of ERCP

ERCP is primarily reserved for cases in which MRCP findings are inconclusive, or when there is a strong clinical suspicion of PSC in patients with IBD despite normal MRCP results. ERCP should be avoided as a routine surveillance tool for disease progression or cancer development due to its inherent risk of complications. Instead, it should be performed in symptomatic patients exhibiting evidence of disease progression, either through worsening cholestatic indices or radiologic deterioration [53].

In this context, particular attention should be directed toward the evaluation and monitoring of dominant strictures, best demonstrated by MRI. The optimal follow-up strategy for PSC remains a matter of debate. A recent study compared three surveillance approaches: annual MRI, on-demand ERCP, and elective ERCP performed at predetermined intervals, and demonstrated that patients undergoing elective ERCP had a lower incidence of cholangiocarcinoma compared with those managed with the other strategies. The primary composite endpoint included liver transplantation, development of hepatobiliary malignancy, or liver-related death. The proportion of patients reaching the composite endpoint was significantly lower in the elective ERCP group (14.1%) compared with the annual MRI group (22.8%) and the on-demand ERCP group (28.2%) [78].

ERCP remains an integral component of the diagnostic work-up in PSC, particularly in cases with suspicious strictures, as it not only increases the diagnostic yield but also allows for therapeutic interventions to relieve biliary obstruction [3].

Beyond its diagnostic applications, especially in the evaluation of indeterminate biliary strictures through cytobrushing, and probe-based confocal laser endomicroscopy (pCLE), cholangioscopy, and targeted biopsies, the primary therapeutic indications for ERCP include the management of symptomatic strictures secondary to bile duct obstruction and recurrent cholangitis [79]. In fact, symptomatic strictures frequently benefit from ERCP-based interventions such as bile duct dilation or stenting, which can provide both symptomatic and biochemical improvement [3]. However, a multicenter randomized trial failed to demonstrate the superiority of short-term stenting over balloon dilation [80].

ERCP also plays a valuable role in the detection of dysplasia, particularly in patients for whom liver transplantation is considered a potential therapeutic option [26]. In all patients with suspected perihilar or distal CCA, ERCP with biliary brushings for cytology and fluorescent in situ hybridization analysis should be obtained [53].

However, ERCP is associated with a non-negligible risk of procedure-related adverse events. In a systematic review and meta-analysis focused on PSC, the overall risk of post-ERCP pancreatitis was 4.2%, whereas bleeding and perforation occurred in only 0.3% and 0.7% of procedures, respectively [81].

#### 3.2.7. Non-Invasive Prognostic Tools

Beyond laboratory tests, imaging, and ERCP, several PSC-specific non-invasive prognostic tools have been developed [82].

The Mayo risk score combines age, bilirubin, AST, albumin and prior variceal bleeding to predict 4-year mortality but was derived mainly in advanced, transplant-referral cohorts and performs best in late-stage disease [29,82].

The primary sclerosing risk estimate tool (PREsTo) model uses hepatic decompensation as the primary outcome (variceal bleeding, ascites, or hepatic encephalopathy); it consists of nine variables: bilirubin, albumin, serum ALP times the upper limit of normal (ULN), platelets, AST, hemoglobin, sodium, patient age, and the number of years since PSC was diagnosed [82,83].

UK-PSC scores use age, bilirubin, alkaline phosphatase, albumin, platelets, extrahepatic biliary disease and variceal hemorrhage to estimate the combined endpoint of liver transplantation or death at 2 and 10 years [82,84].

The Amsterdam-Oxford model uses PSC subtype, age at diagnosis, albumin, platelets, AST, alkaline phosphatase and bilirubin to estimate PSC-related death and/or liver transplant [82,85].

Enhanced liver fibrosis (ELF) score is a non-invasive test that measures three circulating markers of hepatic matrix metabolism: hyaluronic acid, tissue inhibitor of metalloproteinases-1, and pro-peptide of type III procollagen, where higher scores are associated with shorter survival [82]. In patients with PSC, the ELF score, when compared to LSM, may have superior reliability for risk stratification [86]. Table 1 gives a brief overview of the main models/prognostic scores used in clinical practice.

### 3.3. Challenges and Areas of Uncertainty

#### 3.3.1. Low Diagnostic Accuracy for Early Detection of Cholangiocarcinoma

The longitudinal growth of cholangiocarcinoma makes early diagnosis difficult, and the development of new molecular tools for diagnosis is an important priority [87,88,89]. Moreover, the 0.5–1.2% yearly incidence is too low to justify surveillance programs [9,15]. The EASL suggests surveillance with ultrasound and/or MRI/MRCP for CCA and gallbladder malignancy at least yearly in patients with large duct PSC regardless of disease stage, while every 6 months in the presence of cirrhosis [26]. Additionally, a cohort study found that routine monitoring was linked to a substantially higher 5-year survival (68%) compared with no monitoring (20%) [90]. Cholangiocarcinoma remains one of the major clinical challenges in the management and prognosis of patients with PSC. While most patients eventually progress to end-stage liver disease and cirrhosis, approximately 20% will develop cholangiocarcinoma. Early diagnosis of cholangiocarcinoma is particularly challenging, as there is still no clearly defined optimal diagnostic strategy for its timely detection. This malignancy is characterized by aggressive biological behavior and a dismal prognosis [91]. Over the years, several endoscopic modalities have been explored to improve diagnostic accuracy, including cytology, fluorescence in situ hybridization (FISH), probe-based confocal laser endomicroscopy, and single-operator cholangioscopy with targeted biopsies [92]. However, the diagnostic performance of these techniques, when used individually, remains suboptimal. Consequently, early detection and optimal management of cholangiocarcinoma in PSC typically rely on the combined use of multiple complementary modalities. A meta-analysis investigating the sensitivity and specificity of various endoscopic techniques demonstrated that biliary brush cytology has a sensitivity of 52% and a specificity of 99%. FISH, in cases of trisomy, showed a sensitivity of 32% and a specificity of 72%. Probe-based confocal laser endomicroscopy exhibited a sensitivity of 78% and a specificity of 75%, while cholangioscopy showed a sensitivity of 65% and a specificity of 97% [93].

Regarding surveillance for cholangiocarcinoma, serum CA 19-9 is widely used as an adjunct marker, however, its diagnostic accuracy depends highly, and in fact reported sensitivity and specificity vary widely across studies. In fact, EASL Clinical Practice Guidelines on sclerosing cholangitis from 2022, do not recommend using Ca 19-9 for surveillance purposes [26].

The cutoff used and on fucosyltransferases (FUTs) genotype play a role in these differences [53].

For example, with the same cutoff (20 U/mL), the sensitivity and specificity depend whether it is combined with MRI/MRCP (sensitivity of 100% and 38% specificity) or ERPC (100% sensitivity and 43% specificity) [94,95].

Clinically relevant false positives may occur in the setting of cholangitis, biliary obstruction, and other benign hepatobiliary conditions [26,53,96]. Conversely, false negatives can occur, including in individuals who do not express CA 19-9 (e.g., Lewis antigen–negative status) [97,98]. Therefore, CA 19-9 should not be used as a stand-alone test but rather interpreted in the clinical context and combined with imaging and, when indicated, endoscopic tissue sampling [76,95,97].

Despite these efforts, the limited diagnostic sensitivity and specificity, together with the intrinsic aggressiveness of the disease, resulting in a 5-year survival below 10% [78].

#### 3.3.2. Risk of Colorectal Cancer

The risk of colorectal cancer should not be underestimated in patients with PSC, as these individuals often develop cancer at a more advanced stage at a younger age, and with dysplastic lesions that are challenging to detect.

The association between IBD and PSC is well established. A 2021 meta-analysis including 64 studies and 776,700 patients reported the presence of PSC in 2.16% of individuals with IBD. This prevalence was higher among patients with indeterminate colitis compared with those with ulcerative colitis or Crohn’s disease, 5.0% versus 2.4% and 0.9%, respectively [8]. The actual co-occurrence of IBD and PSC might be even higher, in fact it has been shown that the prevalence of PSC in previously diagnosed patients with IBD was three times higher than what had been estimated based on symptoms [99].

Patients with concomitant PSC and IBD have an approximately twofold higher risk of developing advanced-stage colorectal cancer compared with those with IBD but without PSC (37% vs. 22%, *p* = 0.035). Most dysplastic lesions are in the right colon, tend to be multifocal, and are often difficult to detect endoscopically due to their atypical appearance [100].

Both the American (AASLD) and European (EASL) guidelines recommend endoscopic surveillance with a high-quality colonoscope at time of diagnosis for everyone, every 1–2 years in patients with IBD, and for patients with no IBD diagnosis, a colonoscopy is recommended every 5 years [26,53]. Although there is an indication to perform systematic mucosal sampling with four-quadrant biopsies every ten centimeters throughout the colon, this recommendation is supported by low-quality evidence [101]. Further efforts are therefore needed to improve the early detection and diagnosis of dysplastic lesions in the colons of patients affected by PSC.

#### 3.3.3. Risk of HCC and Gallbladder Polyps

Hepatocellular carcinoma (HCC) in patients with PSC is relatively uncommon, with previous studies reporting an incidence of approximately 2.4% during a median follow-up period of 10 years. Current clinical guidelines recommend ultrasound-based surveillance every six months in patients with cirrhosis, and could be tailored according to age, being HCC uncommon in patients under the age of 50 years [102]. Ultrasonography appears to be the most sensitive modality for the detection and evaluation of polyps in the gallbladder, demonstrating higher accuracy compared with MRI for their detection. An optimal size cutoff of approximately 8 mm has been proposed for diagnostic and clinical decision-making purposes [26,103].

#### 3.3.4. Indications for Liver Transplantation

PSC accounts for up to 15% of all liver transplantations. Liver transplantation in patients affected by PSC represents a therapeutic option for individuals with end-stage liver cirrhosis complicated by portal hypertension, for those with recurrent cholangitis, and for those suffering from intractable pruritus. Furthermore, in Europe, the presence of high-grade dysplasia constitutes an indication for liver transplantation [104]. In the United States, liver transplantation is also considered in cases of hilar cholangiocarcinoma, provided that the tumor measures less than 3 cm in diameter and there is no evidence of lymph node or distant organ metastases and after a course of chemo-radiotherapy [105].

The Amsterdam-Oxford model for primary sclerosing cholangitis is used, not only to assess the probability of death by PSC, but also the risk of needing liver transplantation [106].

It is well established that liver transplantation in the setting of cholangiocarcinoma is associated with a poorer prognosis, with a 30% reported 3-year survival even for stage I and II; therefore, probably patients with confirmed high-grade dysplasia rather than those with established cholangiocarcinoma should be considered for liver transplant [104,107,108].

Limited data from a single center study including four PSC patients with hilar cholangiocarcinoma, showed promising results via the combination of liver transplantation with Whipple surgery and chemoradiotherapy. Nevertheless, these data needs further corroboration and more patients [109].

Unfortunately, PSC recurs in approximately ¼ of patients within the first ten years following liver transplantation. Although the etiological factors underlying recurrence remain poorly understood, previous studies have demonstrated that a history of colectomy prior to liver transplantation is associated with a protective effect (HR = 0.65). In contrast, the presence of IBD (HR = 1.73), a prior diagnosis of cholangiocarcinoma (HR = 2.42), and older age at the time of transplant (HR = 1.24) have been identified as potential risk factors for recurrence [110].

According to current futility criteria, liver re-transplantation can be offered when survival expectation exceeds 50% at 5 years [104].

In a UNOS/OPTN analysis of 5080 PSC recipients, 5-year graft and patient survival after re-transplantation were overall inferior to primary transplantation; however, when the indication for re-transplantation was disease recurrence (rPSC), outcomes were similar to primary transplantation (graft survival, *p* = 0.45; patient survival, *p* = 0.09). Mechanical ventilation at the time of re-transplantation was the only independent predictor of 30-day outcome in patients with rPSC [111].

Consistently, data from the Nordic Liver Transplant Registry showed that graft and patient survival after re-transplantation were better when the underlying disease was PSC compared with non-PSC etiologies (5-year graft survival 61% vs. 44%, respectively), and that re-transplantation for rPSC was associated with similar or better graft and patient survival and lower 30-day and 5-year mortality compared with a matched comparison cohort [112].

#### 3.3.5. Limitations

Despite our efforts to carry out a comprehensive review of the available scientific evidence in the literature, this non-systematic review has some potential limitations.

First, we only briefly summarize key pathophysiological and molecular concepts, as the manuscript was designed to provide a clinically oriented overview rather than an in-depth basic science discussion. The second limitation is related to the fact that the authors collected literature exclusively from PubMed and therefore did not assess other potential scientific search engines or databases. A third limitation arises from the fact that the study reports values cited in various articles without further in-depth analysis of those data.

In addition, this review does not address in detail the differences between pediatric and adult PSC.

Finally, this literature review may have been influenced by the authors’ perspectives and personal experience.

## 4. Future Perspectives and Research Directions

Future progress will likely rely on a multi-modal approach that integrates clinical phenotyping with imaging, ERCP-based assessment, and molecular profiling to better define individualized surveillance and treatment strategies.

A priority is improving patient stratification, particularly with respect to malignancy risk. The increasing availability of MRCP-based severity scores, together with emerging radiomics approaches (e.g., GLRLM Run Entropy on FS-T2W sequences), may refine risk prediction. In parallel clinical tools leveraging on machine-learning are expected to accelerate novel associations uncovering and potential risk factors [75]. However, robust external validation and careful calibration across heterogeneous populations will be essential before these tools can be incorporated into pragmatic clinical algorithms to guide surveillance intensity, timing of transplant referral, and selection for clinical trials. AI models are also being applied to imaging to overcome operator dependency, variable diagnostic accuracy, and technical complexity, such as digital single-operator cholangioscopy to help categorize biliary strictures into benign or malignant [113,114].

Moreover, patient-derived organoid platforms may offer a valuable route to better study the pathogenesis and to accelerate preclinical therapeutic screening [115]. In the longer term, these approaches may also open regenerative perspectives, at least conceptually, for repairing or replacing damaged biliary epithelium [116].

Finally, disease-modifying medical therapy remains an unmet need. Continued progress will require well-designed, collaborative multicenter trials and well-selected endpoints to match targeted interventions to the patients most likely to benefit.

## 5. Conclusions

Primary sclerosing cholangitis (PSC) remains a significant diagnostic and therapeutic challenge for clinicians involved in the management and long-term follow-up of affected patients. The principal limitation in advancing knowledge and treatment lies in the low prevalence of the disease, which hampers the conduct of large-scale studies capable of generating high-level evidence and mitigating the inherent underpowering of observational research. PSC exerts a profound impact on both life expectancy and quality of life, owing to its propensity to progress to hepatic failure and cirrhosis, as well as its strong association with an increased risk of cholangiocarcinoma. Further investigations are urgently needed—both to elucidate the pathogenic mechanisms underlying disease onset and progression, thereby facilitating the development of targeted therapeutic strategies, and to refine methods for the early detection of preneoplastic lesions amenable to surgical resection or liver transplantation prior to malignant transformation.

## Figures and Tables

**Table 1:** Non-invasive prognostic models/scores. Figure legend: Bil = bilirubin, AST = aspartate aminotransferase, ALP = alkaline phosphatase, PLT = platelets, Hb = hemoglobin, Liver Tx = Liver Transplant.

Model	Main Endpoint	Variables
Mayo risk score	4-year mortality	Age, Bil., AST, albumin and prior variceal bleeding
PSC risk estimate tool (PREsTo)	Hepatic decompensation	Bil., albumin, ALP, PLT, AST, Hb, Na, Age, the number of years since PSC was diagnosed
UK-PSC scores	Liver Tx or death at 2 and 10 years	Age, Bil., ALP, albumin, PLT, extrahepatic biliary disease and variceal hemorrhage
Amsterdam-Oxford model	Death and/or Liver Tx	PSC subtype, Age, albumin, PLT, AST, ALP, and Bil.
Enhanced liver fibrosis (ELF) score	Tx-free survival, liver-related outcomes	Hyaluronic acid, tissue inhibitor of metalloproteinases-1, and pro-peptide of type III procollagen,

## Data Availability

No new data were created or analyzed in this study.
